# Jak-2 mutation frequency in patients with thrombocytosis

**DOI:** 10.22088/cjim.9.2.189

**Published:** 2018

**Authors:** Osman Yokus, Habip Gedik

**Affiliations:** 1 Department of HematologyMinistry of Health İstanbul Training and Research Hospitalstanbul, Turkey; 2 Department of Infectious Diseases and Clinical MicrobiologyMinistry of Health Bakırköy Sadi Konuk Training and Research Hospitalstanbul, Turkey.

**Keywords:** Thrombocytosis, Janus kinase 2, Essential thrombocytosis

## Abstract

**Background:**

We aimed to investigate the etiologic causes and the existence of Janus kinase 2 mutation (JAK2) in cases with thrombocytosis.

**Methods:**

In this retrospective study, patients who were admitted to hematology clinic with thrombocytosis between 2013 and 2015 were investigated in terms of the etiological causes of thrombocytosis and the existence of JAK2 mutation.

**Results:**

We retrospectively evaluated 136 cases that underwent JAK2 mutation analysis due to ET preliminary diagnosis in our hematology clinic. The mean age of the patients was 56.7±15.3 years (range: 22-86 years) and 71 (52%) were males. JAK2 mutation was found to be positive in 58 (42%) of cases. The mean platelet counts of the cases were 860.25×10^9^ / L (range: 455-1,105 10^9^ /L) and the mean spleen vertical length was 135.1±21.6 mm (range: 110-220 mm).

**Conclusions:**

JAK2 mutation analysis and bone marrow biopsy are the two main procedures to diagnose primary thrombocytosis in adults with persistent thrombocytosis after excluding the causes of secondary thrombocytosis. Myeloproliferative neoplasms and essential thrombocytosis are the common causes of primary thrombocytosis in adults with persistent thrombocytosis, as myelodysplastic syndrome rarely causes primary thrombocytosis.

Thrombocytosis is seen as a primary disease (primary myeloproliferative diseases) or a reactive (secondary) to diseases of the bone marrow (1). Infections, severe iron deficiency, neoplasm, hemorrhage, hemolysis, inflammatory conditions, splenectomy, and some medications cause reactive thrombocytosis (1). Chronic myeloproliferative disorders (CMDs) progress with proliferation in one or more hematopoietic cell populations accompanied with differentiation and maturation. Those include chronic myeloid leukemia (CML), polycythemia vera (PV), essential thrombocytosis (ET) and primary myelofibrosis (PMF) (2). The CML is distinguished from others by the presence of Philadelphia (Ph) chromosome t (9:22) or BCR-ABL molecule, as PV, ET and PMF are called as Ph chromosome negative myeloproliferative disorders (3). Janus kinase 2 (JAK2) mutation was discovered in 2005 in Ph chromosome negative myeloproliferative disorders and was found in the whole of PV cases and about 50% of the ET and PMF cases (4, 5). According to the classification of the World Health Organization (WHO) in 2008, JAK2 positivity was accepted to be one of the major criteria in the diagnosis of Ph-negative myeloproliferative disorders (6). JAK2 gene is also used as a diagnostic marker. It has been reported that the JAK2 mutation frequency in ET is about 50% and about 60% in all myeloproliferative disorders (MDs) (7). 

There are not enough studies about the frequency of JAK2 mutation in ET and thrombocytosis cases yet (2). In this study, we aimed to investigate the etiologic causes of thrombocytosis and the existence of JAK2 mutation in cases with thrombocytosis, retrospectively.

## Methods

In this retrospective study, the patients, who were admitted with thrombocytosis (platelet count > 450×10^9^ /L) that remained for at least three months, and followed up at the Hematology clinic of Ministry of Health Istanbul Training and Research Hospital between 2013 and 2015, were investigated in terms of the etiologic causes of thrombocytosis and existence of JAK2 mutation patients. The patients were evaluated based on patient files and hospital records. 

All causes of primary and secondary thrombocytosis were investigated. Patients initially underwent baseline tests such as a complete blood count, sedimentation, peripheral blood smear, CRP, ferritin, autoimmune disease tests (ANA, anti-DNA, RF etc.), Brucella tube agglutination test, ELISA (anti-HIV, anti-HCV, anti-HBs), urinalysis, stool blood test, cancer markers (CA 19-9, CEA, PSA), abdominal ultrasonography, chest x-ray, endoscopy, JAK2 mutation and Ph chromosome for the differential diagnosis of primary and secondary thrombocytosis. 

The etiologic causes of reactive thrombocytosis and clonal neoplasms other than ET have not been evaluated in our study. Infection-related thrombocytosis was generally referred as "inflammatory" and detailed. Diseases such as rheumatoid arthritis and systemic lupus were also referred as autoimmune diseases without reference to subgroups.

SPSS Version15.0 for Windows program was used for the statistical analysis. Descriptive statistics were described as the number and percentage for the categorical variables; mean, standardized, minimum, maximum for the numerical variables. 

Mann-Whitney U test was used for independent numerical comparisons between the two groups that did not satisfy the normal distribution condition. The Kruskal Wallis test was performed because the independent group-to-group comparisons were not provided for the normal distribution condition of numerical variables. Subgroup analyses were performed by Mann-Whitney U test and interpreted by Bonferroni correction. The ratios of categorical variables between the groups were tested by chi-square analysis. Statistical significance levels of alpha were accepted as p<0.05.

## Results

We retrospectively evaluated 136 cases that underwent JAK2 mutation analysis due to thrombocytosis as a preliminary diagnosis in the hematology clinic. The mean age of the patients was 56.7±15.3 years (range: 22-86 years) and 71 (52%) were males. 

The means age of males was 56.4±15.6 years and of females was 56.9±15.2 years, respectively. There was no statistically significant difference between males and females in terms of mean age (P=0.970). 

Bone biopsy was performed in 106 (77.9%) cases for the definite diagnosis. JAK2 mutation was found to be positive in 58 (42%) of cases. JAK2 mutation was found to be more frequent in males. The mean platelet counts of all cases were 860.25×10^9^ / L (range: 455-1,105 10^9^ /L), and the mean spleen vertical length was 135.1±21.6 mm (range: 110-220 mm). 

Primary thrombocytosis consisted of 75% of the cases in the etiology. The distribution of the diagnosis groups was ET in 59 (43%) cases, policytemia vera in 19 (14%) cases, inflammation in 18 (13%) cases, primary myelofibrosis in 9 (6%) cases, myeloproliferative neoplasms (MPNs) in 9 (6%) cases, iron deficiency anemia (IDA) in 8 (5%) cases, splenectomy in 5 (3%) cases, CML in 3 (2%) cases, myelodysplastic syndrome (MDS) +MPN in 2 (1%) cases, malignancy in 2 (1%) cases, MDS in 1(1%) cases, and autoimmune disease in 1 (1%) case. 

The mean age of the cases with JAK2 mutation (p: 0.004), the mean of spleen vertical lengths (p=0.004) and percentage of bone biopsy (p: 0.001) were found to be significantly higher than those without the JAK2 mutation. ET, PV, and PMF were recorded more frequently in patients with JAK2 mutation, whereas reactive causes (inflammation, DEA, splenectomy) were recorded predominantly in cases without JAK2 mutation. The frequency of diagnosis among the groups was found to be significantly different (p= 0.001, table 1). 

**Table 1:** Relationship between the presence of JAK-2 mutation and variable of cases with thrombocytosis

	**JAK2 (+)** **n: 58**	**JAK 2 (-)** **n:78**	**P-value**
Age (Mean±SD) years	60.7±15.9	53.7±14.2	0.004
**Gender**			
Male n (%) Female n (%)	24 (41.4) 34 (58.6)	41 (52.6) 37 (47.4)	0.197
platelet count (x 10^9 ^/ L)	952±147	792.025±235	0.765
Vertical spleen length (Mean±SD)	130.9±20.6	139.8±22.0	0.004
**Biopsy**			
Performed Non-performed	58 (54.8) 0 90.0)	48(45.2) 30 (100)	<0.001
**Definite diagnosis**			
Essential thrombocytosis Inflammatory Iron deficiency anemia Myeloproliferative neoplasms (MPN) Splenectomy Primary myelofibrosis Policytemia vera Chronic myeloid leukemia Malignancy Myelodysplastic syndrome (MDS) MDS + MPN Autoimmune diseases	33 (56.9) 0 (0.0) 0 (0.0) 3 (5.2) 0 (0.0) 5 (8.6) 16 (27.6) 0 (0.0) 0 (0.0) 0 (0.0) 1 (1.7) 0 (0.0)	26 (33.3) 18 (23.1) 8 (10.3) 6 (7.7) 5 (6.4) 4 (5.1) 3 (3.8) 3 (3.8) 2 (2.6) 1 (1.3) 1 (1.3) 1 (1.3)	<0.001

## Discussion

Primary thrombocytosis and ET are more frequent in women and older ages. Clinically frequent thrombotic attacks, basal hemorrhage, splenomegaly, megakaryocytic hyperplasia in the bone marrow can be observed in ET (8). Our findings suggest that patients with thrombocytosis in older age should be investigated in terms of primary thrombocytosis, as reactive thrombocytosis is less common in those ages. On the other side, the causes of reactive thrombocytosis, such as iron deficiency anemia and autoimmune diseases, are more common in the middle-aged women and also JAK2 mutation is negative. In recent years, JAK2 mutation has been a major criterion for CMDs and facilitated the differential diagnosis. The JAK2 mutation frequency was reported to be below 1% in the normal population, whereas it was 62.2% in CMD cases; 97.6% in PV cases; 54.5% in ET cases and 53.44% in PMF cases (9, 10). ET, of which its annual incidence is 1-2 in 100.000 and was observed more commonly in the 60s, had the highest frequency of JAK2 positivity in our study. Approximately 50% of ET patients were reported to have JAK2 mutation, greater blood cell counts and more complications than cases with JAK2 negative (11). 

JAK2 mutation was reported to be positive in 73% of PV patients, in 61% of ET cases, and in 55% of PMF cases in that study. The frequencies of JAK2-V617F mutation were reported to be 80% in the PV cases and 42 % in the ET cases in a study from Turkey. Leukocyte counts, hemoglobin values and the presence of splenomegaly were significantly different in patients with JAK2 mutation (12). In a study including 140 cases with CMDs, the rates of JAK2 mutation were found to be 82% (23.28) in PV cases, 53.1% (17.32) in ET patients, 40% (4.10) in PMF cases, and 60% (6.10) in other indistinguishable CMDs cases, respectively (7). In another study conducted in the 108 cases with CMDs cases in Taiwan by Liu et al, JAK2 mutation was found to be positive in 85% (28.33) PV cases, 59% of patients (29.49) in ET patients, 33% (2.6) in PMF cases, (0.11) in MDS patients, and (0.10) in the other cases with 10 different hematological diseases (13). In our study, the frequency of JAK2 mutation in the cases and mean age of the patients are lower than those in other studies. JAK2 mutation is more common in older adults and they have a higher risk of thrombosis (10). The mean of spleen vertical lengths was greater in cases with JAK2 mutation in our study, as JAK2 mutation was reported to be more common in ET patients with organomegaly (13). The frequency of the JAK2 mutation was reported to be insignificant between men and women with thrombocytosis in a study from Turkey (14). It was reported that there is a relationship between the presence of JAK2 mutation and risk factors related to complications, such as advanced age at diagnosis, presence of the higher leukocyte count, itching, organomegaly, and larger spleen sizes (15, 16). 

This is more likely associated between the JAK2 mutation and splenomegaly. However, there was not a relationship found between the presence of JAK2 mutation and splenomegaly in cases with BCR‑ABL negative CMDs in a study from Turkey (14). Since cases with thrombocytosis were assessed only in terms of the frequency of JAK-2 mutation in that study and the frequencies were found to be different from those in other studies that assessed the frequency of JAK-2 mutation in cases with BCR‑ABL‑negative CMDs (12, 17). The limitation of our study is that a limited number of cases with thrombocytosis were evaluated in terms of JAK2 mutation, retrospectively. 

There is a need to study greater number of cases from different hematology clinics to describe the exact frequency of JAK2 in cases with primary thrombocytosis from Turkey. However, our findings were different from other studies conducted in Turkey. It is obvious that the frequency of JAK2 mutation and other risk factors related to complications may vary by country and study population. The prospective and big populations include studies needed to be defined appropriately.

JAK2 mutation analysis and bone marrow biopsy are two main procedures to diagnose primary thrombocytosis in adults with persistent thrombocytosis after excluding the causes of secondary thrombocytosis. Myeloproliferative neoplasms and essential thrombocytosis are the common causes of primary thrombocytosis in adults with persistent thrombocytosis, as myelodysplastic syndrome rarely causes primary thrombocytosis. The ratios of primary and secondary ET cases in the etiology of thrombocytosis vary by age and hematology clinic where the study is conducted. JAK2 mutation analysis and then a bone marrow biopsy might be a reasonable way to confirm the definite diagnosis after assessment of the characteristics of patients with thrombocytosis and causes of reactive thrombocytosis. 
